# Divergent effects of vitamins K1 and K2 on triple negative breast cancer cells

**DOI:** 10.18632/oncotarget.26765

**Published:** 2019-03-19

**Authors:** Sarah Beaudin, Leila Kokabee, JoEllen Welsh

**Affiliations:** ^1^ Cancer Research Center and Department of Environmental Health Sciences, University at Albany, Rensselaer, NY 12144, USA

**Keywords:** vitamin K, menaquinone, phylloquinone, γ-carboxylation, triple-negative breast cancer

## Abstract

Vitamin K serves as an essential co-factor in the γ-carboxylation of glutamate to γ-carboxyglutamate (GLA), a post-translational modification mediated by gamma-glutamyl carboxylase (GGCX) and vitamin K oxidoreductases (VKORC1 or VKORC1L1). While both phylloquinone (K1) and menaquinone (K2) support the synthesis of GLA-modified proteins, studies assessing K1 and/or K2 effects in cancer cells have reported minimal effects of K1 and anti-proliferative or pro-apoptotic effects of K2. qPCR results indicated highest expression of *GGCX*, *VKORC1*, and *VKORC1L1* in triple negative breast cancer (TNBC) cell lines, Hs578T, MDA-MB-231 and SUM159PT, and in advanced stage disease. To assess differential effects of vitamin K, TNBC cells were cultured in media supplemented with K1 or K2. K1 treatment increased cell growth, and enhanced stemness and GLA-modified protein expression in TNBC lysates. Alternatively, lysates from cells exposed to vehicle, K2, or the VKOR antagonist, warfarin, did not express GLA-modified proteins. Further, K2 exposure reduced stemness and elicited anti-proliferative effects. These studies show that TNBC cells express a functional vitamin K pathway and that K1 and K2 exert distinct phenotypic effects. Clarification of the mechanisms by which K1 and K2 induce these effects may lead to relevant therapeutic strategies for manipulating this pathway in TNBC patients.

## INTRODUCTION

Vitamin K compounds are essential nutrients that are well-characterized for their ability to promote blood coagulation through post-translational modifications to clotting factors. The only unequivocal function of vitamin K is to act as cofactor for the enzyme gamma-glutamyl carboxylase (GGCX) which catalyzes the gamma (γ) carboxylation of glutamate residues to γ-carboxyglutamate (GLA) residues. Carboxylation by GGCX is coupled to oxidation of vitamin K, and maintenance of this reaction requires one of two vitamin K epoxide reductases (VKORs) to continuously regenerate the reduced form of vitamin K. The vertebrate genome encodes two *VKOR* genes: *VKORC1* (primarily expressed in liver, lung, and exocrine tissues including mammary gland) and *VKORC1L1* (expressed in brain). Both enzymes support reduction of vitamin K and GGCX activity *in vitro* and *in vivo*, however their tissue-specific functions are poorly understood [1, 2]. The VKORs are the targets of vitamin K antagonists such as warfarin [3], with VKORC1 more sensitive to inhibition by warfarin than VKORC1L1.

In proteins that are γ-carboxylated, this modification is critical for function, in part because the GLA residues impart high-affinity calcium (Ca++) binding [4]. Although the best characterized GLA proteins are clotting factors, others function in bone, including osteocalcin (also known as bone GLA protein and encoded by the *BGLAP* gene) and matrix gla protein (MGP). While < 20 γ-carboxylated proteins have been identified to date, the presence of GGCX and VKORs in a wide variety of tissues suggests more extensive physiological and pathological roles for γ-carboxylation. Emerging studies have indeed linked GGCX GLA carboxylations to lung, bladder, and prostate cancer [5–8]. GLA modification of GAS6, a ligand for the TAM (TYRO, AXL, MERTK) family of receptors, has been linked to smooth muscle cell proliferation, neural stem cell survival, and pancreatic cancer progression [9–11]. Periostin, an extracellular matrix component strongly linked to cancer progression, was recently identified as a γ-carboxylated protein in a screen of mesenchymal stromal cells [12]. For these newly recognized GLA proteins, the functional consequences of γ-carboxylation have yet to be fully explored.

The biology of vitamin K is complex and its role in cancer is understudied. Naturally occurring compounds that reverse coagulation defects due to dietary deficiency include phylloquinone (K1; present only in plant foods) and menaquinone (K2; present in fermented foods, meats, and dairy products). Both forms can support the *in vitro* synthesis of GLA proteins required for coagulation and bone homeostasis, but their transport, cellular uptake, and metabolism differ, leading to tissue-specific effects [13–16]. The few studies that have assessed effects of K1 or K2 in cancer cells typically report minimal effects of K1 and anti-proliferative or pro-apoptotic effects of K2 [17–21]. The caveat to published work is that only one study [17] directly compared K1 and K2 in a breast cancer cell line (BC-M1 cells) and that study reported effective concentrations for growth inhibition at mM doses, well above the physiological (nM) ranges. Complicating the interpretation of these data is evidence that K2 can exert γ-carboxylation independent effects through the SXR nuclear receptor [22, 23] and that K1 and K2 may enhance intracellular antioxidant pathways critical to cell survival [24].

To gain insight into the potential impact of the vitamin K pathway in breast cancer, we annotated expression of *GGCX*, *VKORC1*, and *VKORC1L1*, profiled GLA protein modifications, and directly compared the effects of K1 and K2 in multiple breast cancer model systems. Our data demonstrate that the vitamin K pathway leading to γ-carboxylation is functional in triple negative breast cancer (TNBC) cells, and that vitamins K1 and K2 exert distinctly different effects on their phenotype.

## RESULTS

### Relevance of vitamin K pathway in breast cancer

**Figure 1:**
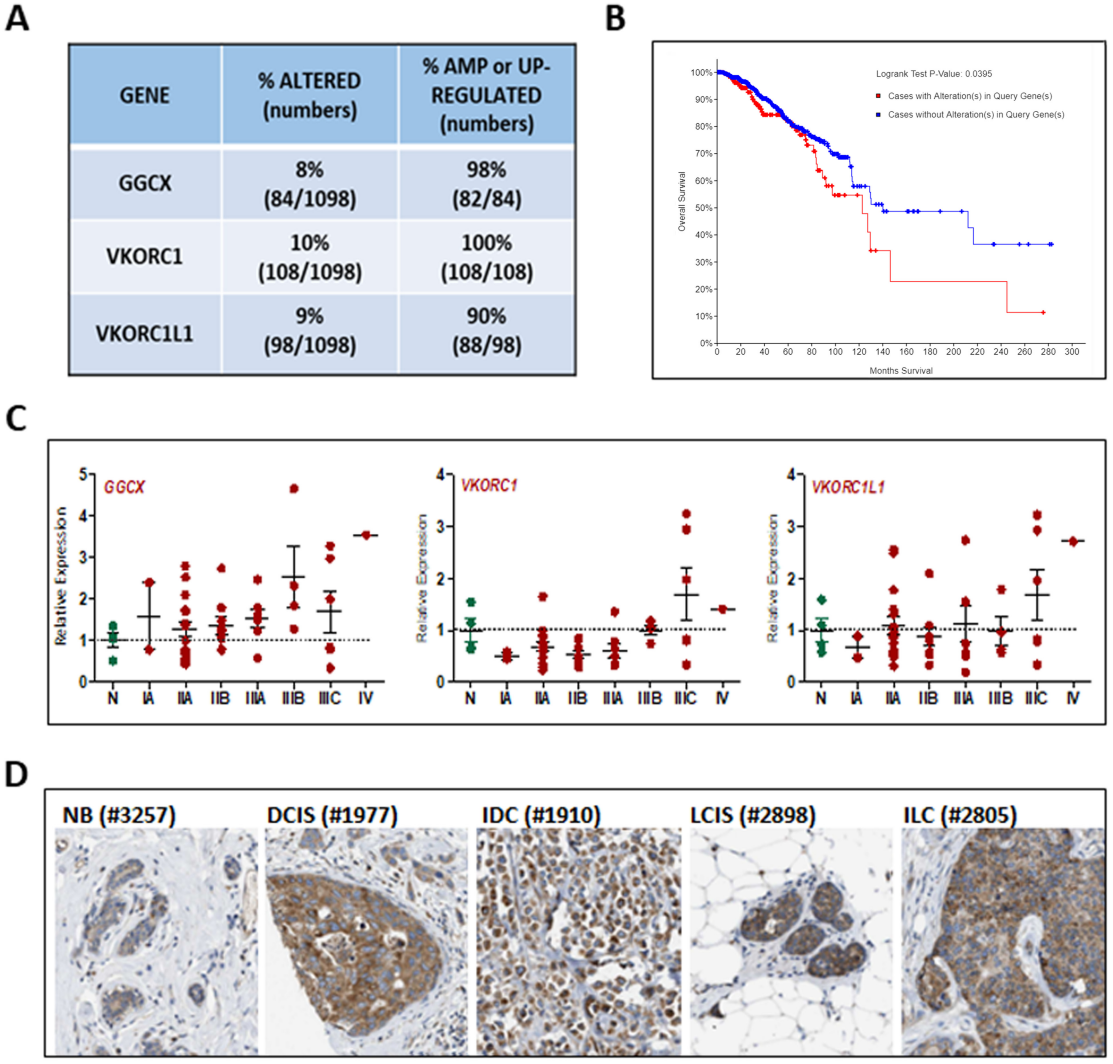
Relevance of vitamin K pathway to human breast cancer **(A)** Analysis of genomic alterations in *GGCX*, *VKORC1*, and *VKORC1L1* genes obtained from the TCGA dataset of 1098 breast cancers. The following alterations were included: mutations based on exome sequencing, copy number alterations based on the GISTIC (Genomic Identification of Significant Targets in Cancer) algorithm, and mRNA z-scores based on RNA-Seq data (threshold ± 2). **(B)** Kaplan Meier analysis indicated reduced median survival of patients whose tumors harbor mutations in *GGCX*, *VKORC1*, and *VKORC1L1*. **(C)** *GGCX*, *VKORC1*, and *VKORC1L1* expression in human breast tumor tissue samples. TissueScan™ Disease Tissue qPCR Arrays (#BCRT104, Origene) were used to assess gene expression in 48 samples (4-normal, 2-Stage IA, 15-Stage IIA, 9-Stage IIB, 7-Stage IIIA, 4-Stage IIIB, 6-Stage IIIC, 1-Stage IV). Data was normalized to *β-Actin* expression and expressed relative to values obtained from normal tissue (N). Bars represent mean ± SEM. **(D)** GGCX protein expression in human breast tumors. Images of tissues immunostained with validated antibody against GGCX (Atlas Antibodies HPA018284) from the Human Protein Atlas [25]. NB-normal breast, DCIS-ductal carcinoma *in situ*, IDC-invasive ductal carcinoma, LCIS-lobular carcinoma *in situ*, ILC-invasive lobular carcinoma. Numbers in parentheses refer to deidentified patient codes.

All three genes involved in the vitamin K cycle (*GGCX*, *VKORC1*, and *VKORC1L1*) are expressed in normal mouse mammary gland [21] yet there is no published data regarding their expression in human breast or breast cancer. To explore the relevance of γ-carboxylation in breast cancer, we first analyzed datasets from The Cancer Genome Atlas (TCGA) and found that approximately 25% of 1098 invasive breast cancers exhibit amplification or up-regulation of *GGCX*, *VKORC1*, and/or *VKORC1L1* (Figure 1A). More importantly, the overall survival of patients whose tumors highly expressed one or more of these genes is significantly reduced compared to those whose tumors do not (Figure 1B). Using TissueScan arrays representing 4 normal tissues and 44 breast cancers (Figure 1C), we confirmed up-regulation of *GGCX* and *VKORC1L1* in a subset of tumors beginning as early as Stage IIA. Up-regulation of *VKORC1* was less frequent but was detected in some late stage tumors. Publicly available data on the Human Protein Atlas [25] confirm that GGCX protein is expressed in normal breast epithelium and that both *in situ* and invasive ductal and lobular breast tumors express the enzyme at high levels (Figure 1D). Staining for GGCX was localized only in tumor cells indicating that stromal cells are unlikely to contribute to protein γ-carboxylation. Collectively, the available genomic and proteomic data suggest that the vitamin K-dependent pathway genes, *GGCX*, *VKORC1*, and *VKORC1L1,* are present in normal mammary gland but up-regulated in a subset of invasive breast cancers that are characterized by poor overall survival. Because GGCX-mediated γ-carboxylation requires vitamin K, these data support the concept that vitamin K status has clinical relevance for breast cancer patients.

### Expression of vitamin K pathway in breast cancer model systems

**Figure 2:**
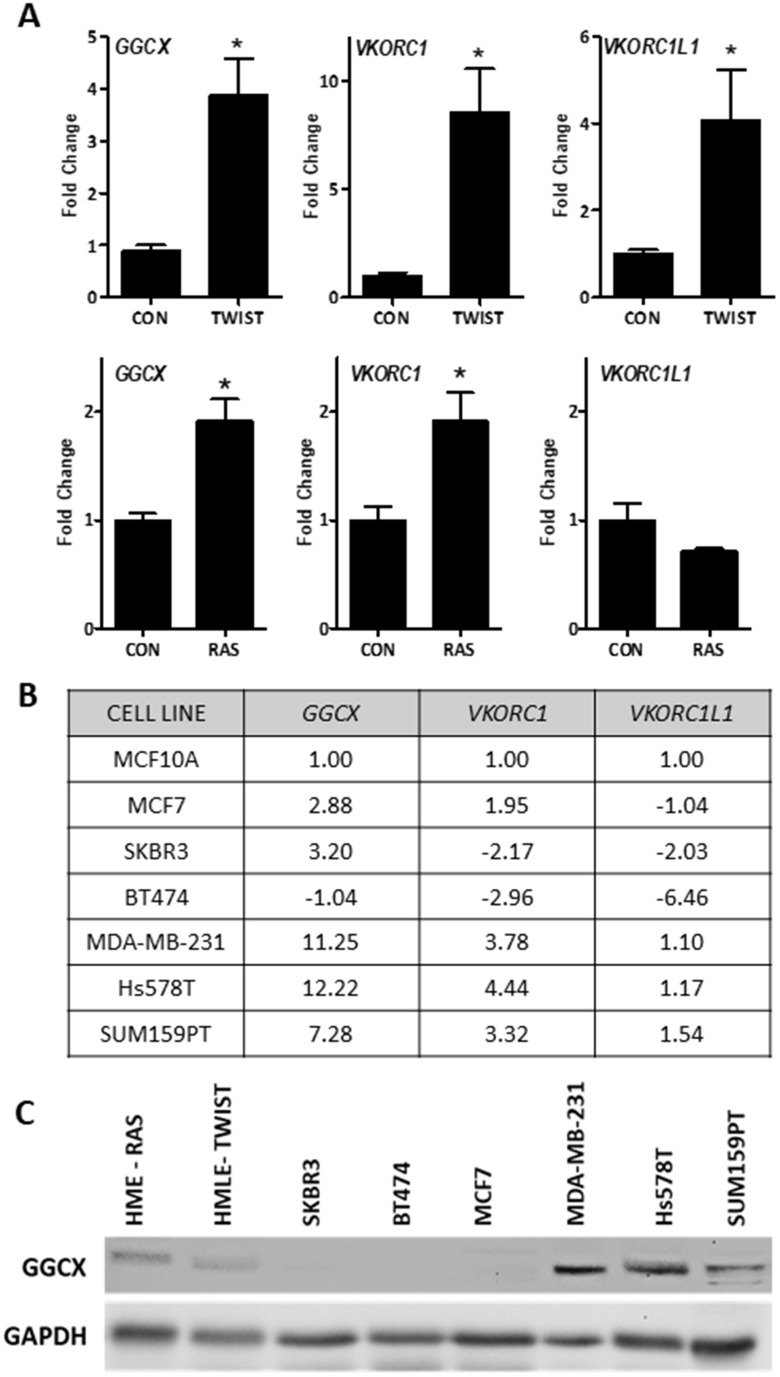
Basal expression of the vitamin K pathway in cellular models of breast cancer **(A)** Expression of *GGCX*, *VKORC1*, and *VKORC1L1* in mammary epithelial cells expressing TWIST (top) or RAS (bottom). **(B)** Summary table of gene expression in non-tumorigenic breast cells (MCF10A) and breast cancer cell lines: ER+ (MCF7), HER2+ (SKBR3, BT474), and triple negative (MDA-MB-231, Hs578T, and SUM159PT). For *A-B*, data was normalized to *18S* and expressed relative to control cells (*A*-HMLE or HME, *B*-MCF10A) set to 1. Fold-change values represent mean of 3 independent biological replicates run in duplicate. *A* shows fold change ± SD. *Significantly different compared to control (p < 0.05) as measured by one-way ANOVA and Tukey post-test. **(C)** Western blot for GGCX and GAPDH loading control in breast cancer cell lines and mammary epithelial cells expressing RAS (HME-RAS) or TWIST (HMLE-TWIST).

To mechanistically address the role of vitamin K and γ-carboxylation in breast cancer, we screened a panel of breast cancer cell models for *GGCX*, *VKORC1*, and *VKORC1L1* expression. *GGCX* and one or more *VKOR* genes were up-regulated in estrogen receptor-negative (ER-) mammary epithelial cells expressing the epithelial mesenchymal transition transcription factor TWIST, or the oncogene RAS (Figure 2A). We also examined the expression of these vitamin K pathway genes in a panel of breast cancer cell lines (Figure 2B, Supplementary Table 1). *GGCX* and *VKORC1* were highly upregulated in TNBC cells MDA-MB-231, Hs578T, and SUM159PT relative to MCF10A or non-TNBC cells. Western blotting (Figure 2C) confirmed that GGCX is expressed in all three TNBC cell lines as well as in mammary epithelial cells expressing RAS or TWIST, but is undetectable in HER2+ (SKBR3, BT474) or luminal (MCF7) cells.

### Effects of vitamin K on breast cells *in vitro*

**Figure 3:**
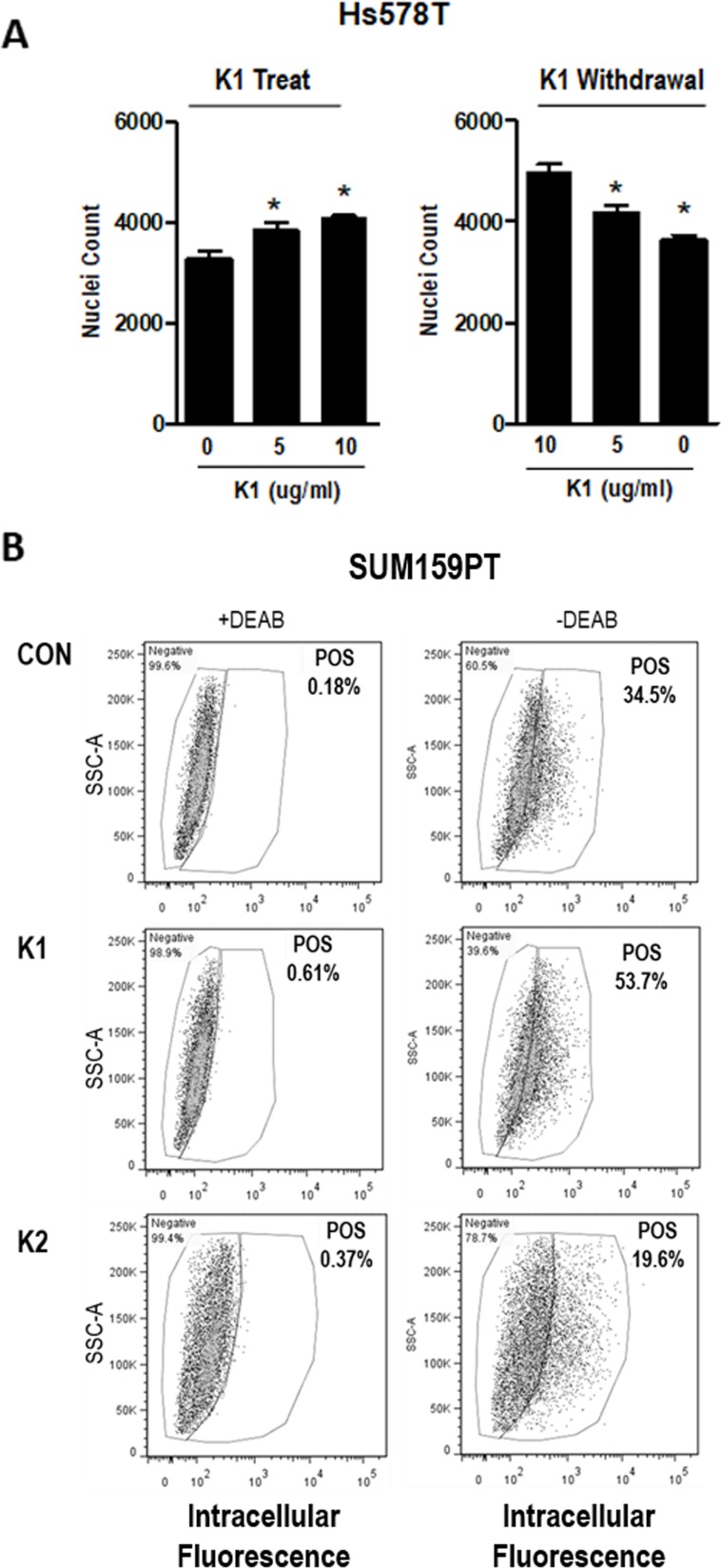
Effect of K vitamins on TNBC cell density and ALDH+ population **(A)** *Left*, Nuclei counts of Hs578T cells grown in standard media and plated with 0, 5, or 10 μg/ml K1 for 72 h growth assay. (A) *Right*, Cells were maintained in K1 (5 μg/ml) for > 3 passages and plated with 0, 5, or 10 μg/ml K1 for 72 h growth assay. Data represents mean ± SD of 3 biological replicates run in triplicate. *Significantly different compared to control (p<0.05) as measured by one-way ANOVA and Tukey post-test. **(B)** SUM159PT cells were maintained in media containing vehicle (CON), 5 μg/ml K1, or 5 μg/ml K2 and assayed with the ALDEFLUOR™ kit (STEMCELL Technologies). Cells were assessed ± DEAB (negative control). POS-percentage of cells that are ALDH positive.

As an indicator of VKOR activity in breast cancer cells, the effects of vitamin K treatment were assessed. Since standard cell culture media does not contain vitamin K, we supplemented media of breast cancer cells with phylloquinone (vitamin K1). Addition of 5 or 10 μg/ml K1 (approximately 10-20 μM) to Hs578T cells (Figure 3A, left panel) dose-dependently increased cell density, although the effect was modest. When cells were maintained in K1 for several passages and re-plated in media without K1, growth decreased (Figure 3A, right panel). Similar results were observed in SUM159PT cells (Supplementary Figure 1).

Given the TCGA data indicating that expression of vitamin K pathway genes correlates with poor survival in breast cancer patients, we hypothesized that GGCX activation leading to γ-carboxylation would promote aggressive phenotypes including acquisition of stem cell properties. We therefore tested whether K1 altered the percentage of cells positive for aldehyde dehydrogenase 1 (ALDH1), a validated breast cancer stem cell marker. SUM159PT cells grown in media supplemented with K1 (5 μg/ml) or vehicle for 48 h were analyzed with the ALDEFLUOR™ assay kit (STEMCELL Technologies). Per manufacturer’s instructions, samples were prepared in duplicate adding an inhibitor of ALDH enzyme activity, DEAB, to one of the duplicates to serve as a negative control. The data from the DEAB-containing sample was used to set the gates to differentiate ALDH+ and ALDH- cells within each treatment. As shown in Figure 3B, K1 supplementation induced a marked increase in the percentage of ALDH+ cells relative to control cultures (53.7% vs. 34.5% Pos). Although Hs578T cells are known to express most stem cell features, they do not typically express ALDH [26]. Consistent with this, we detected a low percentage (4%) of ALDH positivity in vehicle-treated Hs578T cells and no measurable difference upon exposure to K1 (data not shown).

**Figure 4:**
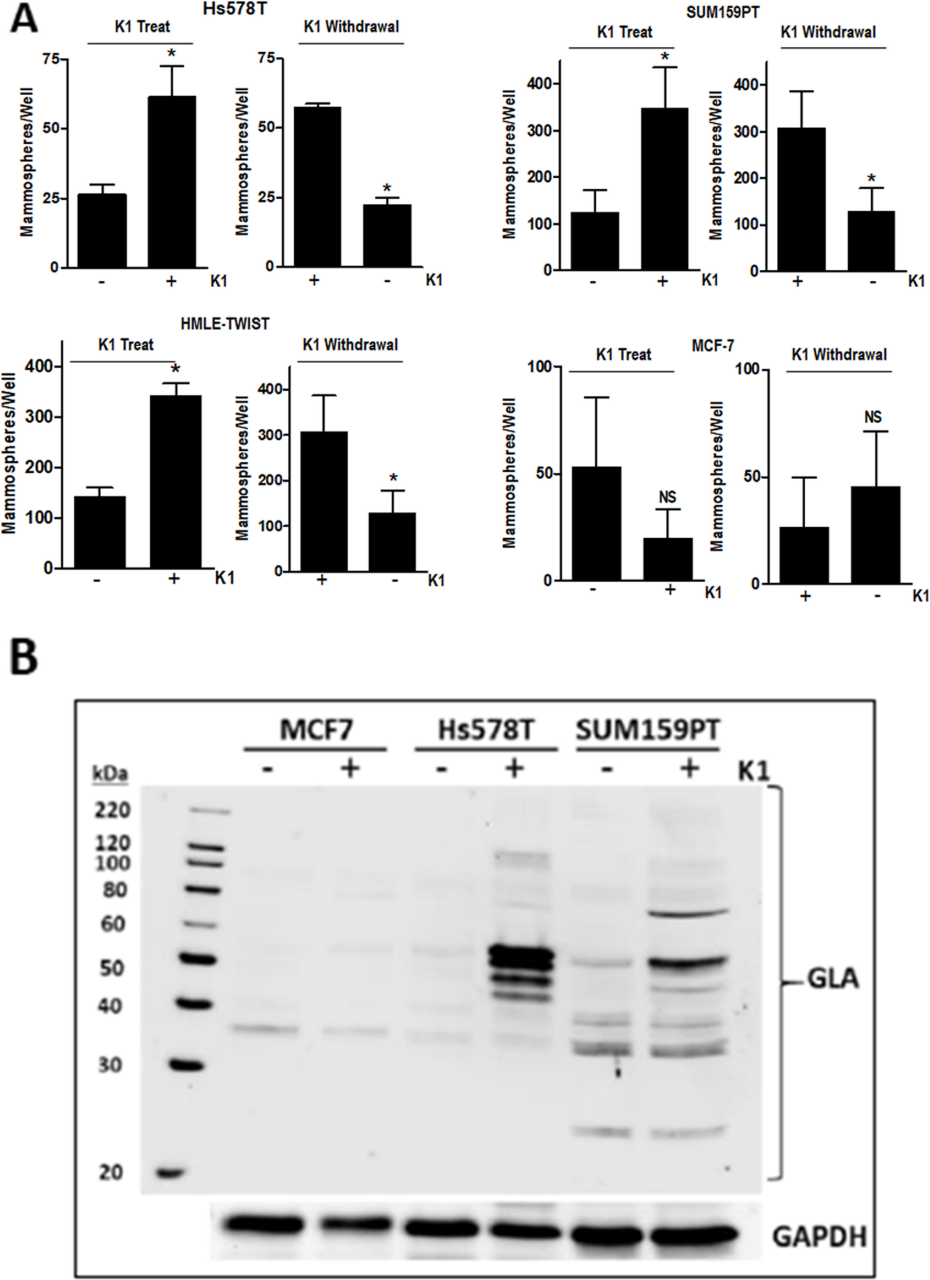
Effect of vitamin K1 treatment and withdrawal on mammosphere formation and GLA protein expression **(A)** For K1 treatment studies, Hs578T, SUM159PT, HMLE-TWIST, and MCF7 cells were grown in standard media and plated in ultra-low attachment plates in Mammocult™ media (STEMCELL Technologies) in the presence or absence of 5 μg/ml K1. For K1 withdrawal studies, cells were maintained in media supplemented with 5 μg/ml K1 for > 3 passages and then plated in Mammocult™ media with or without 5 μg/ml K1. For both treatment and withdrawal studies, mammospheres were imaged and counted after 8 days. Bars represent mean ± SD of 2-3 biological replicates conducted in triplicate for all cell lines except HMLE-TWIST (one biological replicate conducted in triplicate). *p < 0.05, NS-not significant. **(B)** MCF7, Hs578T, and SUM159PT cells were cultured in media containing 5 μg/ml K1 or ethanol vehicle for > 3 passages. Whole cell lysates were analyzed by western blotting with antibodies against GLA or GAPDH as loading control. Blot is a representative of at least 4 blots from independent samples with similar results.

To further assess the effect of K1 on stemness, we performed two types of mammosphere protocols. First, we tested the effect of K1 addition to mammosphere media during sphere formation. Second, we tested the effect of K1 withdrawal during sphere formation for cells that had been passaged in K1. For both protocols, cells were plated in low-attachment plates containing Mammocult™ media and incubated for 8 days. As shown in Figure 4A, Hs578T, SUM159PT, and HMLE-TWIST cells maintained in standard media (ie, lacking K1) displayed enhanced mammosphere-forming capacity when K1 was present in Mammocult™ media (“K1 treat”). Furthermore, when these three cell lines were maintained in K1 media and plated in Mammosphere™ media lacking K1 (“K1 withdrawal”), sphere formation decreased. In contrast, K1 had no consistent effect on mammosphere formation of MCF-7 cells (Figure 4A), and MCF10A cells did not form spheres in these assays even if maintained in K1 (not shown).

### Vitamin K1 promotes synthesis of γ-carboxylated proteins in TNBC cells

Promotion of the stem cell phenotype by K1 has not previously been reported for any cell types, thus the underlying mechanisms are unknown. Because the best described function for vitamin K is as cofactor for GGCX, we assessed whether supplementing culture media with K1 would promote γ-carboxylation in TNBC cells. As a general screen for proteins post-translationally modified by GGCX, we utilized an antibody that specifically recognizes γ-carboxyglutamate (GLA) residues. Lysates from cells grown for 48 h in media with vehicle or K1 (5 μg/ml) were separated on SDS-PAGE gels and blotted with a pan α-GLA antibody (Sekisui Diagnostics). As shown in Figure 4B and Supplementary Figure 2, unique patterns of GLA-modified proteins were induced in Hs578T, SUM159PT, and MDA-MB-231 cells supplemented with K1. Importantly, GLA modifications were also induced by K1 in mammary epithelial cells expressing TWIST but not in MCF-7 cells or in MCF10A cells (Figure 4 and data not shown).

### Comparative effects of K1, K1-EPO, and K2 *in vitro*

**Figure 5:**
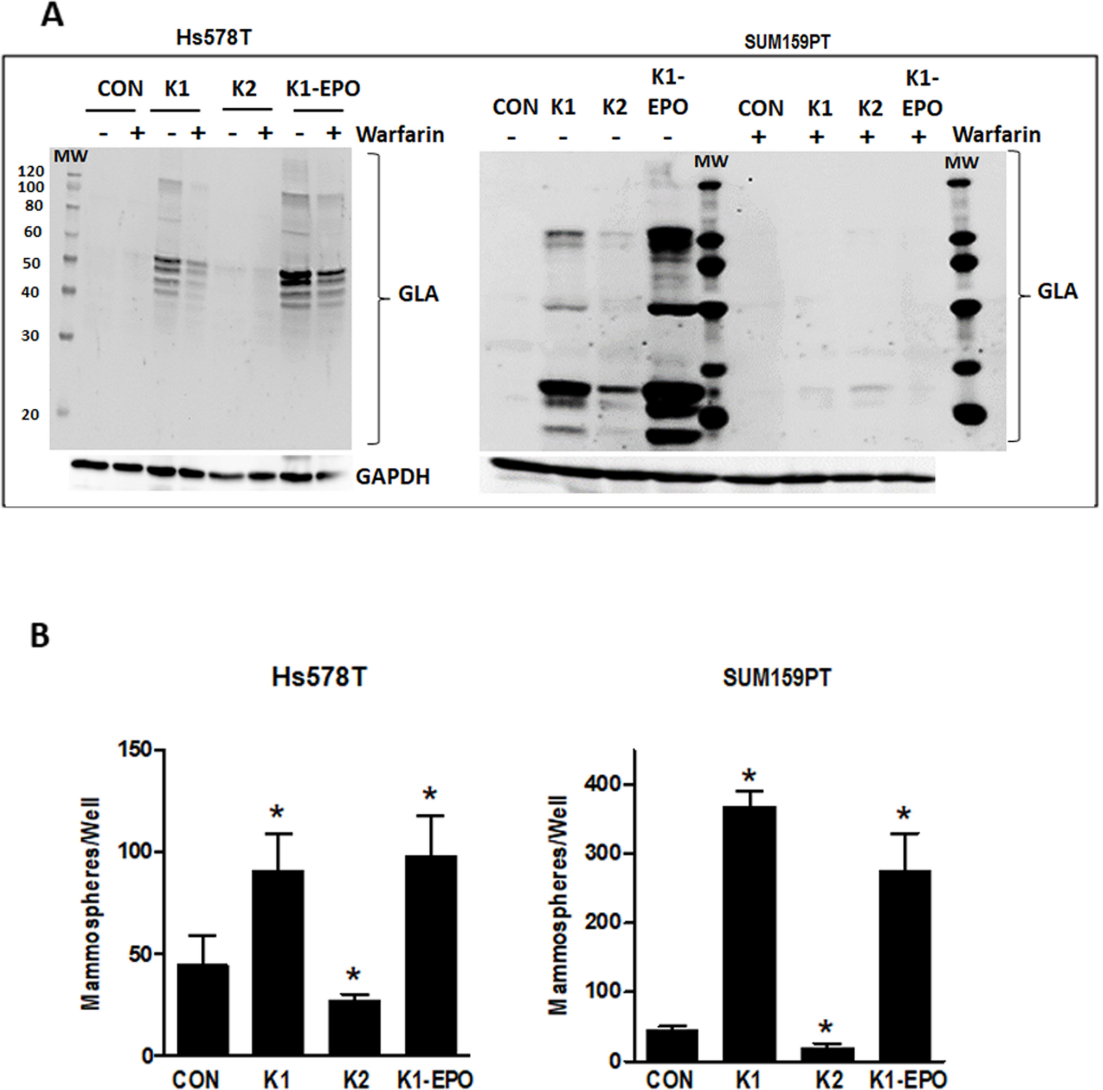
Effects of vitamins K2, K1-EPO, and VKOR inhibitor on GLA protein expression and mammosphere formation in TNBC cells **(A)** Hs578T (left) and SUM159PT (right) cells were maintained in media containing ethanol vehicle or 5 μg/ml K1, K2, or K1-EPO for > 3 passages. Post-attachment, cells were switched to media ± K1, K2, K1-EPO, and 2 μM warfarin for 48 h. Whole cell lysates were analyzed by western blotting for GLA or GAPDH as loading control. Blots are representative of at least 3 independent samples with similar results. **(B)** Hs578T (left) and SUM159PT (right) cells were grown > 3 passages in standard media containing EtOH vehicle or 5 μg/ml K1, K2, or K1-EPO and plated in ultra-low attachment plates in Mammocult™ media (STEMCELL Technologies). After 8 days, mammospheres were imaged and counted. Bars represent mean ± SD of 3 biological replicates analyzed in triplicate. *Significantly different from CON (p < 0.05) as measured by one-way ANOVA and Tukey post-test.

As noted earlier, the two forms of vitamin K (K1 and K2) cycle between reduced and oxidized forms during γ-carboxylation. The data reported in Figures 3-4 reflect the effects of K1 in its reduced form. We next explored the effects of the 2,3-epoxide form of K1 (K1-EPO) in these assays. Since K1-EPO is only active as cofactor for GGCX after reduction, we expected this compound to mimic the effects of K1 if cells express functional VKORC1 or VKORC1L1. Indeed, K1-EPO induced similar patterns of GLA protein modifications as K1 and promoted mammosphere formation in both Hs578T and SUM159PT cells (Figure 5). Also, as expected, GLA modifications induced by either K1 or K1-EPO were inhibited in cells co-treated with the VKOR inhibitor, warfarin (Figure 5, Supplementary Figure 2). These data confirm that TNBC cells express functional VKORC1 and/or VKORC1L1 complexed with functional GGCX and that both reduced and oxidized forms of K1 promote stemness in TNBC. We next tested vitamin K2, which is known to promote γ-carboxylation as efficiently as K1 in cell-free assays. Surprisingly, the reduced form of K2 had minimal effects on γ-carboxylated protein synthesis in TNBC cell lines (Figure 5A). Furthermore, K2 suppressed rather than promoted mammosphere formation in both SUM159PT and Hs578T cells (Figure 5B) and reduced the ALDH+ population below that of control cells (Con: 34.5%; K2: 19.6%) (Figure 3B).

**Figure 6:**
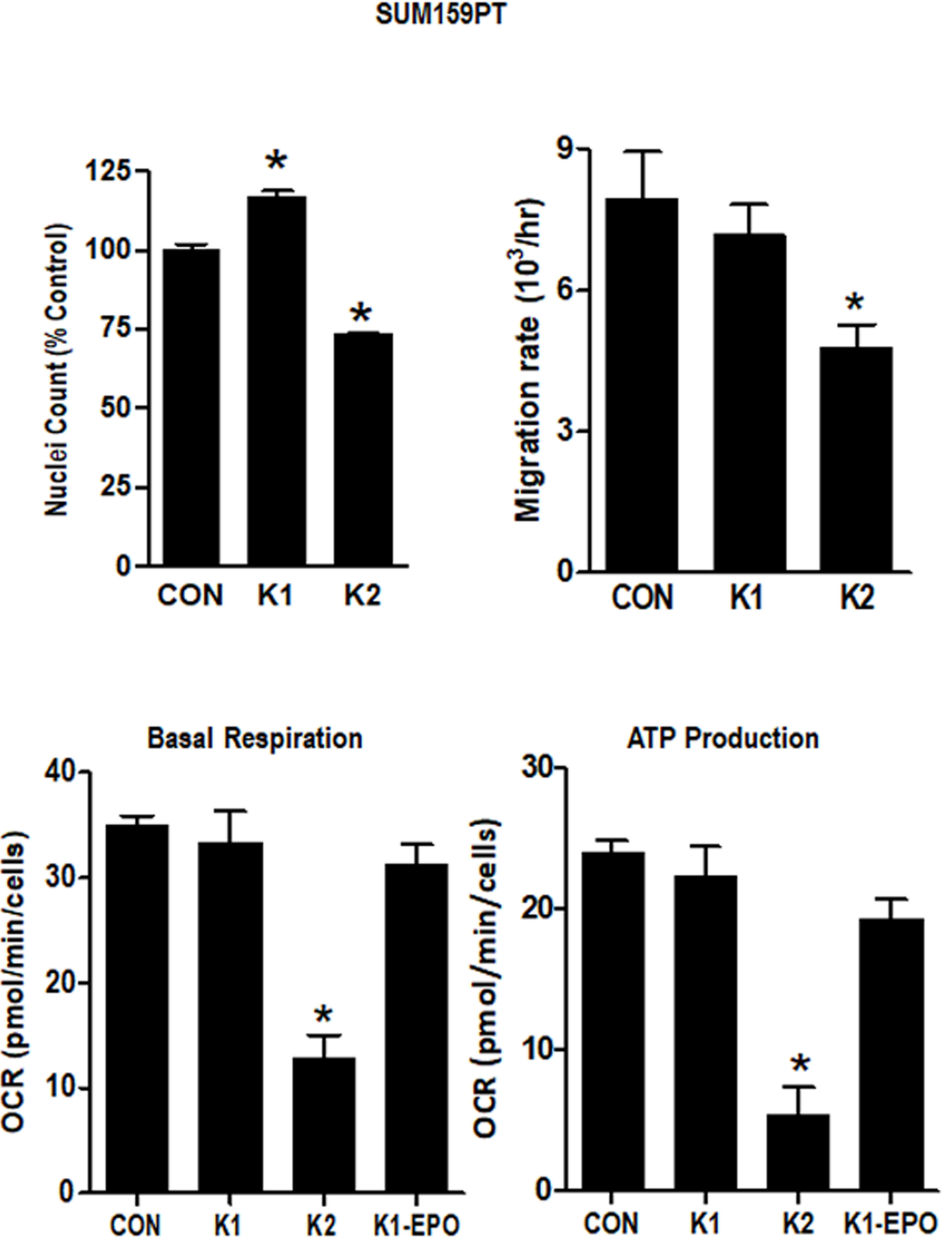
Effect of vitamins K1 and K2 on nuclei counts, migration rates and metabolism in SUM159PT cells Cells were maintained in standard media supplemented with EtOH vehicle or 5 μg/ml K1, K2, or K1-EPO for > 3 passages. For nuclei count, cells were formaldehyde-fixed, incubated with Hoechst, and imaged on the IN Cell Analyzer. For migration assays, confluent monolayers were scratched and imaged live on an EVOS live cell imaging system (ThermoFisher Scientific). For metabolism assays, cells were analyzed with the Seahorse XF Cell Mito Stress Test (Agilent). Basal Respiration and ATP Production were calculated with WAVE software (Agilent) after normalization by DNA content. Bars represent mean ± SD of 3 technical replicates. *Significantly different from CON (p < 0.05) as measured by one-way ANOVA and Tukey post-test.

To further evaluate the comparative effects of K1 and K2 on TNBC cells, we evaluated growth, migration, and mitochondrial metabolism in SUM159PT cells. As shown in Figure 6, K2 suppressed adherent cell growth (measured as nuclei counts) and migration (measured after wounding of confluent monolayers) whereas K1 increased nuclei count and had no significant effect on migration. Using the Seahorse extracellular flux analyzer, we found that K2 reduced basal respiration and ATP production and increased basal ECAR whereas K1 and K1-EPO had no effects on these metabolic parameters (Figure 6, Supplementary Figure 3). Collectively, these data support the concept that K2 and K1 exert distinct effects on breast cancer cells, with K1 (and its epoxide) promoting aggressive phenotypes and K2 suppressing growth, migration, and mitochondrial metabolism.w

## DISCUSSION

There is consensus that the dietary guidelines for vitamin K, which were developed based on its role in promoting hepatic γ-carboxylation for optimal coagulation, require revision based on newly recognized functions in extrahepatic tissues [27]. In this regard, the historical view that K1 and K2 are functionally equivalent also requires re-assessment [4, 28]. The major finding of the studies reported here is that the two forms of vitamin K exert distinct effects on breast cancer cells, with K1 promoting γ-carboxylation and stem cell features, and K2 suppressing growth and energy metabolism. Interestingly, K1 increased GGCX activity and promoted synthesis of γ-carboxylated proteins in TNBC cells but not in normal mammary epithelial cells or hormone responsive breast cancer cells. This observation is consistent with our finding that vitamin K pathway genes required for γ-carboxylation (*GGCX*, *VKORC1*, and *VKORC1L1*) were highly expressed in TNBC cell lines compared to non-TNBC cells. In addition, TCGA data indicated that the subset of patients whose breast tumors exhibit genomic amplification or up-regulation of *GGCX*, *VKORC1*, and *VKORC1L1* had significantly reduced overall survival. Collectively, these findings suggest increased activity of the canonical vitamin K pathway with more aggressive cancer phenotype.

In association with γ-carboxylation, supplementation of TNBC cell media with K1 increased cell numbers, enriched for ALDH+ cells, and promoted mammosphere formation. Because both K1 and its 2,3-epoxide coordinately enhanced γ-carboxylation and mammosphere formation whereas K2 had minimal effects on γ-carboxylation and did not promote stem cell features, it seems likely that these phenotypic changes are casually related to γ-carboxylation. However, further studies are necessary to mechanistically test this suggestion and to determine the nature of the γ-carboxylated proteins produced by TNBC cells supplemented with vitamin K1.

Previous studies have demonstrated differences in uptake and metabolism of K1 and K2 in established cell lines (HepG2, MG-63) and primary human fibroblasts [15, 16]. However, stimulation of GLA protein synthesis was equivalent in fibroblasts incubated in K1 and K2 at concentrations as low as 0.1 μM. Thus, it was unexpected that vitamin K2 did not induce γ-carboxylated protein synthesis or mimic the effects of K1 and K1-EPO in breast cancer cells. Because K2 reduced growth, migration, and energy metabolism, the lack of effects of K2 on γ-carboxylation and stem cell markers (ALDH+ and mammosphere formation) was not likely due to impaired cellular uptake, although comparison of cellular K1 and K2 concentrations would be necessary to confirm this. Instead, internalized K2 may be more rapidly catabolized than K1, limiting its accessibility to GGCX in the endoplasmic reticulum and leading to alternative, GGCX-independent effects. In support of the latter, K2 has been shown to alter gene expression *in vitro* and *in vivo* via activation of the nuclear steroid and xenobiotic receptor SXR [23, 29, 30]. SXR is expressed in breast cancer cells, with higher levels reported in estrogen receptor-negative tumors [31]. While SXR can be activated by a range of chemically and structurally diverse ligands in a context dependent manner, the majority of studies indicate SXR has growth stimulatory effects in breast cancer cells [32]. In contrast, our studies in TNBC and other reports in various cancer cell lines (colon, liver, renal, prostate, and glioma) indicate growth suppression by K2 [18, 19, 21, 33–35]. While it remains possible that activation of SXR by K2 in TNBC induces a distinct program of gene expression leading to growth suppression, further studies are necessary to mechanistically test this possibility.

In the only previous study on the effects of K2 on breast cancer cells, Kiely et al [20] demonstrated inhibition of proliferation and adhesion in MDA-MB-231 cells treated with K2, although the IC_50_ was >100 μM (10-fold higher than used in our study). The reason for the discrepancy in concentrations of K2 required to elicit growth inhibitory effects in various cancer cells is unclear. Emerging studies have suggested that K2 binds to apolipoproteins *in vivo* and is unequally distributed in mammalian tissues. A newly discovered enzyme UbiA Prenyltransferase Domain Containing 1 (UBIAD1) has been shown to biosynthesize K2 from menadione and contribute to tissue specific accumulation of K2 pools [28]. Of particular interest, UBIAD1 was previously identified as TERE-1, a tumor suppressor gene originally identified in prostate and bladder cancers [36, 37]. More recently, Fredericks et al [18, 23] linked TERE1 expression in prostate and renal cancer cells to K2 activation of SXR leading to altered mitochondrial metabolism, accumulation of cellular cholesterol, and growth suppression. These data are consistent with our findings that K2 suppresses growth and mitochondrial metabolism in aggressive TNBC cells, but more detailed analysis of UBIAD1 and SXR expression and function in relation to the effects of K2 in these cells are warranted.

Our data raise important questions regarding the relevance of dietary vitamin K for breast cancer patients. While the concentrations used for our *in vitro* studies are supraphysiologic, the accumulation and metabolism of K1 and K2 in normal breast tissue or breast tumors has not been assessed. Thus, further studies are needed to determine which form of vitamin K predominates in breast epithelia – tumor-promoting K1 or tumor-suppressive K2, and whether cellular handling of these two forms of vitamin K changes during tumorigenesis. Both forms of vitamin K accumulate in liver and peripheral tissues such as muscle and fat after oral supplementation [38–41], and K2 can be further metabolized. In addition to UBIAD1, which as noted above prenylates menadione to generate K2 [42], an as yet unidentified enzyme can convert K1 to menadione [43]. Thus, we hypothesize that in normal breast, K1 is converted to menadione which is prenylated by UBIAD1 to K2, favoring tumor suppression. In tumors with loss of UBIAD1, K2 formation is abrogated, leading to accumulation of K1 which promotes aggressive phenotypes via γ-carboxylation if tumors express GGCX. Further studies to evaluate the function of UBIAD1 in relation to cellular K1 and K2 content and GGCX activity in breast cancer cells are ongoing to test this model.

## MATERIALS AND METHODS

### Cell culture

These studies utilized both non-tumorigenic mammary epithelial cells and breast cancer cells representative of disease sub-types. MCF10A cells (ATCC, Manassas, VA) were cultured in DMEM/F12, 5% horse serum, epidermal growth factor (EGF), hydrocortisone, and insulin. HME and HME-RAS cells (HME cells expressing SV40 and oncogenic H-RAS), were obtained from Dr. Robert Weinberg [44] and were maintained in serum-free M171 media plus MEGS (Life Technologies). HMLE cells expressing EMT transcription factor TWIST or GFP (HMLE-TWIST and HMLE-GFP respectively) were obtained from Dr. Sendurai Mani [45] and maintained in 50:50 DMEM/F12 and M171 plus MEGS, insulin, EGF, and hydrocortisone.

Breast cancer cell lines representative of receptor-positive (SKBR3, BT474, MCF7) and triple negative (MDA-MB-231, Hs578T, SUM159PT) disease were also studied. HER2+ SKBR3 cells (ATCC) and triple positive BT474 cells (ATCC) were grown in DMEM hi-glucose with 10% fetal bovine serum (FBS). ER+/PR+ MCF7 cells (ATCC) were cultured in α-medium essential media (α-MEM) supplemented with 5% FBS. MDA-MB-231 (ATCC), were grown in DMEM hi-glucose with 10% FBS; Hs578T (ATCC) were cultured in DMEM hi-glucose with 10% FBS and insulin, and SUM159PT cells (obtained from Dr. Stephen Ethier [46]) were maintained in Ham’s F12 media with 5% FBS, insulin, and hydrocortisone.

Other than trace amounts present in serum, cell culture media do not contain vitamin K. To assess the impact of chronic exposure to vitamin K compounds on breast cells, we supplemented media with 5 μg/ml (∼11 μM) of vitamin K1, vitamin K2, or the 2,3-epoxide form of vitamin K1 (abbreviated K1-EPO). All vitamin K compounds were obtained from Millipore Sigma (St. Louis, MO). Control cells were grown in parallel in media to which equivalent volume of ethanol vehicle was added. The data reported here represents cells maintained for at least 3 passages prior to analyses. All cell lines were maintained in a 37°C and 5% CO_2_ incubator and passaged every 3-4 days. MCF10A, MCF7, SKBR3, BT474, HMLE-TWIST, and MDA-MB-231 cell lines were authenticated in March 2016, SUM159PT and Hs578T cells were authenticated in November 2015, and HME-RAS cells were authenticated November 2016 by the University at Albany Center for Functional Genomics DNA sequencing facility using the short tandem repeat method.

### Gene expression analyses

Quantitative PCR was used to assess expression of vitamin K pathway related genes in the panel of breast cell lines. RNA was isolated from cells with the Qiagen RNeasy kit (Qiagen, Valencia, CA) and analyzed for concentration and purity on a Nanodrop 1000 Spectrophotometer. cDNA was prepared using Taqman Reverse Transcriptase Reagents (Life Technologies, Grand Island, NY) and analyzed in duplicate using PowerUp SYBR Green PCR Master Mix (Applied Biosystems, Foster City, CA) on a QuantStudio 12K Flex System (Applied Biosystems, Foster City, CA). *GGCX*, *VKORC1*, and *VKORC1L1* primer sequences were obtained from Origene (Rockville, MD) and synthesized by IDTDNA (Coralville, IA). Sequences are listed in Supplementary Table 2. The ΔΔCt method was used for calculations and Ct values were normalized to *18S*. Values presented for cell lines in Figure 2A were expressed relative to relevant control cell lines: HMLE-GFP (top) or HME (bottom). For Figure 2B, data are expressed relative to non-tumorigenic MCF10A cells.

### Western blotting

Cells were plated in 100 mm dishes in media containing K1, K2, K1-EPO, or vehicle and treated with 2 μM warfarin (Millipore Sigma) or DMSO vehicle for 48 h post-attachment. Whole cell lysates were collected in 2x Laemmli buffer, sonicated, and assessed for protein concentration by Pierce BCA protein assay (ThermoFisher Scientific, Waltham, MA). Samples containing 50 μg of protein and 5% β-mercaptoethanol (Millipore Sigma) were separated on SDS-PAGE gels, wet-transferred to PVDF membranes, blocked for 1 h in 5% skim milk/PBS, and incubated overnight with primary antibodies directed against GGCX (Proteintech, Rosemont, IL) at a 1:1,000 dilution or the GLA-domain (Sekisui Diagnostics, Stamford, CT) at a 5 μg/ml dilution in 5% skim milk/PBS/0.1% Tween-20 at 4°C. Blots were then incubated with anti-rabbit or anti-mouse ECL horseradish peroxidase-linked secondary antibody (GE Healthcare, Buckinghamshire, UK) diluted 1:5,000 in 5% skim milk/PBS/0.1% Tween-20 for 1 h. SuperSignal West Dura Extended Duration Substrate (ThermoFisher Scientific) was used for imaging of blots on an iBright CL1000 system (ThermoFisher Scientific). Blots were stripped with acetonitrile, re-probed with GAPDH (AbD Sertotec, Raleigh, NC) primary antibody diluted 1:16,000 overnight, followed by incubation with anti-mouse ECL horseradish peroxidase-linked secondary antibody (GE Healthcare) diluted 1:5,000 for 1 h.

### Measurement of cell density

For nuclei count assays, Hs578T cells were plated at a density of 2,000 cells/well and SUM159PT cells at a density of 5,000 cells/well in 96 well plates in the presence of K1, K2, K1-EPO, or vehicle. Post-attachment, cells were treated with 2 μM warfarin or vehicle. After 72 h, cells were fixed with 4% formaldehyde and incubated with 0.1 μg/ml Hoechst (bisBenzimide H33258, Millipore Sigma) for 30 mins. Plates were read on an IN Cell Analyzer 2200 (GE Healthcare) and data was analyzed with the IN Cell Analyzer 1000 Workstation.

### Mammosphere formation assays

The impact of vitamin K compounds on the stem cell phenotype was assessed with mammosphere assays. Hs578T (6,000 cells/well) and SUM159PT (4,000 cells/well) cells were plated in MammoCult Human Media (STEMCELL Technologies, Vancouver, CA) with added heparin and hydrocortisone in 6-well low attachment plates. Cells were treated with K1, K2, K1-EPO, or vehicle in the presence or absence of 2 μM warfarin. Mammospheres were imaged after 8 days on an IN Cell Analyzer 2200 (GE Healthcare) and counted manually or with NIST’s Integrated Colony Enumerator software (Gaithersburg, MD).

### Disease tissue qPCR arrays

To assess relevance of the vitamin K pathway in human breast tissue, we utilized commercial cDNA arrays. Breast Cancer cDNA Array IV (Origene) plates containing cDNA derived from human tissue were incubated with PowerUp SYBR Green PCR Master Mix (Applied Biosystems) and primers for human *GGCX*, *VKORC1*, and *VKORC1L1.* Primer sequences were obtained from the database at Origene and synthesized by IDTDNA. Plates were run in duplicate for each gene. Gene expression was analyzed on a QuantStudio 12K Flex System (Applied Biosystems). The ΔΔCt method was used for calculations and Ct values for samples were normalized to those of *β-actin*. Values were expressed relative to those of normal breast samples to determine differences in gene expression with increasing stage.

### Aldefluor assay

SUM159PT cells were maintained in 5 μg/ml K1, 5 μg/ml K2, or ethanol vehicle for > 3 passages. ALDH+ populations were assessed on a BD LSR II flow cytometer (BD Biosciences, San Jose, CA) in samples ± DEAB (negative control) using the ALDEFLUOR™ kit from STEMCELL Technologies. BDFACSDiva (BD Biosciences) and FlowJo software (FlowJo, Ashland, OR) were used to calculate the percentage of cells positive for ALDH activity.

### Cell migration assay

SUM159PT cells were cultured > 3 passages in media containing 5 μg/ml K1, K2, or ethanol vehicle. Cells were plated in 6 well plates in standard media containing the same treatments. Each well was uniformly scratched after cells formed a confluent monolayer. Scratches were imaged every 15 mins using an EVOS live cell imaging system (ThermoFisher Scientific). TScratch software (CSE Lab, Swiss Federal Institute of Technology, Zurich, Switzerland) was used to measure scratch area. Rate was calculated as change in scratch area divided by time elapsed.

### Seahorse Bioscience XFp extracellular flux analysis

SUM159PT cells were maintained in 5 μg/ml K1, K2, K1-EPO, or ethanol vehicle for > 3 passages. Cells were plated at a density of 5,000 cells per well in standard media containing the same treatments in Seahorse XFp cell culture miniplates (Agilent, Santa Clara, CA). After attachment, cells were analyzed using the Seahorse XFp Cell Mito Stress Test kit (Agilent) on the XFp Extracellular Flux Analyzer (Agilent). Oxygen consumption rates (OCR) and extracellular acidification rates (ECAR) were measured before and after each drug injection. Basal respiration and ATP production data was calculated using the Seahorse Wave software (Agilent).

### Statistical analysis

Graphpad Prism software (La Jolla, CA) was used for statistical analysis. Data is expressed as mean ± standard deviation of 3 independent experiments. Significance was determined by one-way ANOVA followed by a Tukey post-test. *indicates significant difference (p < 0.05) from control value.

## SUPPLEMENTARY MATERIALS FIGURES AND TABLES






