# Supplementary material for: Divergent effects of vitamins K1 and K2 on triple negative breast cancer cells
Source: Oncotarget. 2019 Mar 19;10(23):2292–305. doi: 10.18632/oncotarget.26765 (PMC6481349; doi:10.18632/oncotarget.26765)
Supplement: Supplementary file 1 [file oncotarget-10-2292-s001.pdf]

Divergent effects of vitamins K1 and K2 on triple negative breast cancer cells

SUPPLEMENTARY MATERIALS

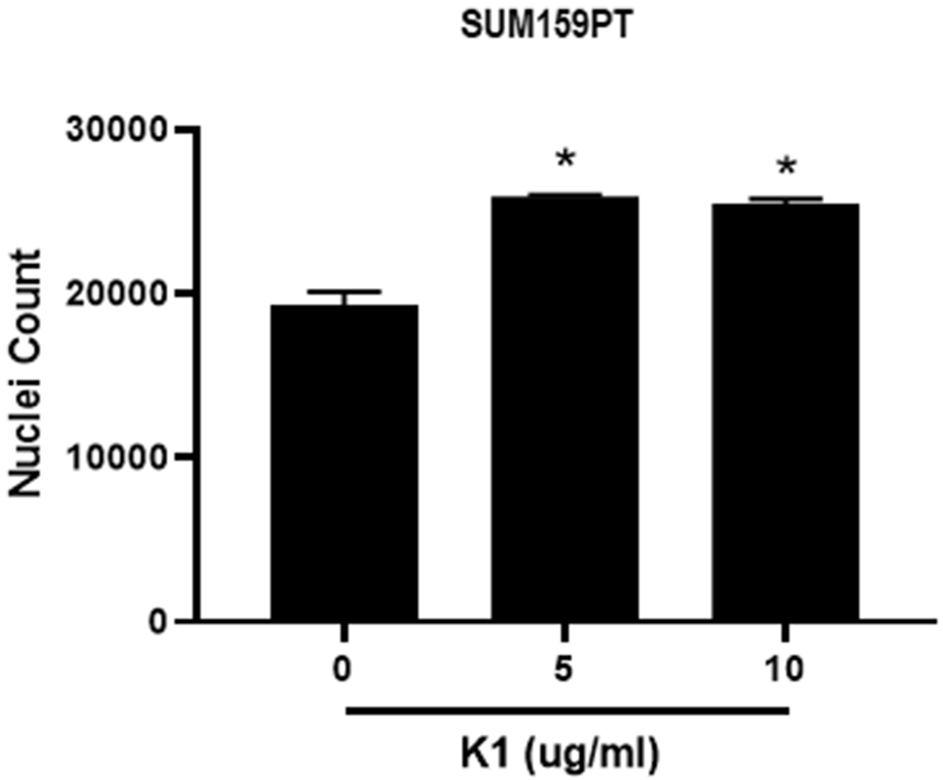

Supplementary Figure 1: Effect of vitamin K1 on SUM159PT cell density. Nuclei counts of cells grown in standard media and plated with 0, 5, or 10 µg/ml K1 for 72 h growth assay. Data represents mean ± SD of 3 biological replicates run in triplicate.

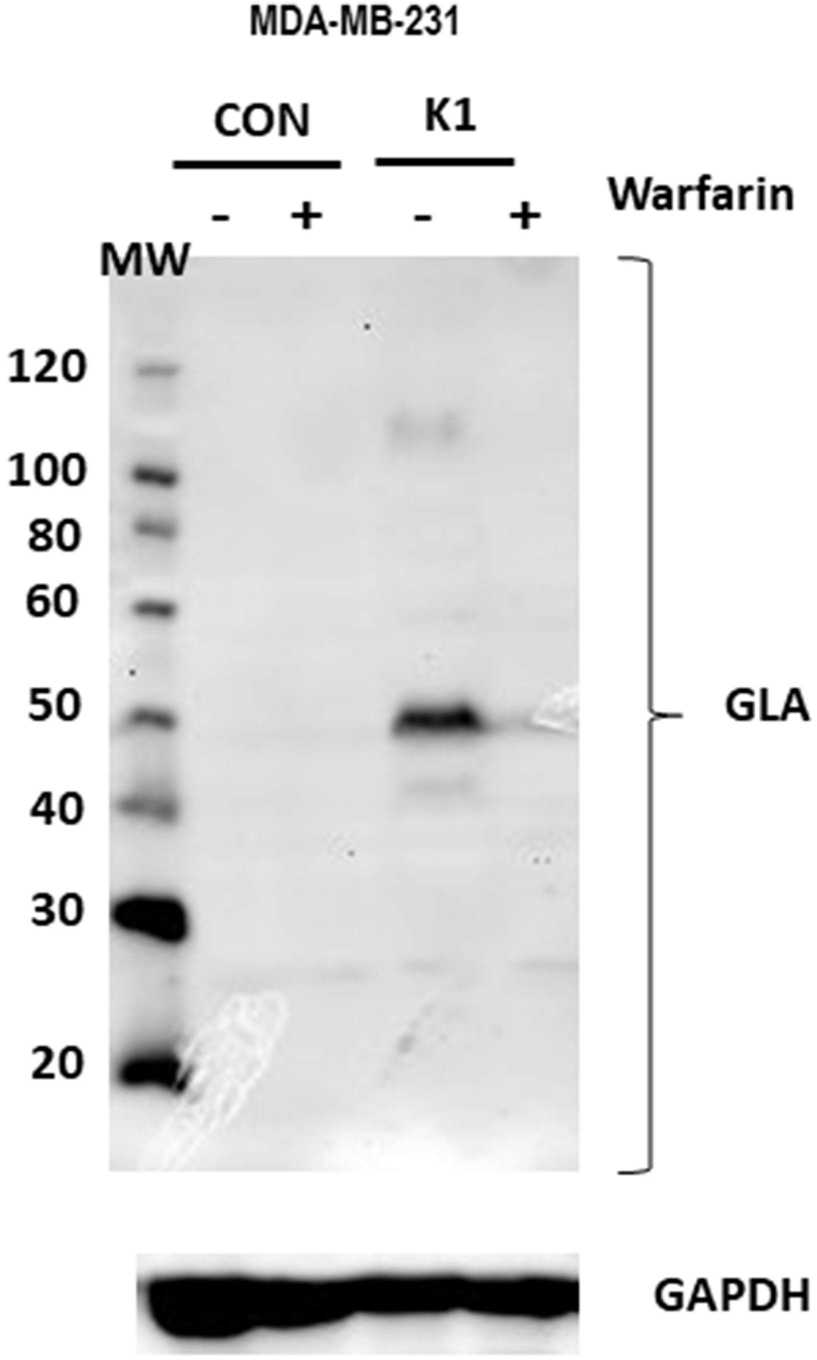

Supplementary Figure 2: Effects of vitamin K1 and a VKOR inhibitor on GLA protein expression in MDA-MB-231 cells. Cells were maintained in media containing ethanol vehicle or 5 µg/ml K1 for > 3 passages. Post-attachment, cells were switched to media ± K1 and 2 µM warfarin for 48 h. Whole cell lysates were analyzed by western blotting for GLA or GAPDH as loading control.

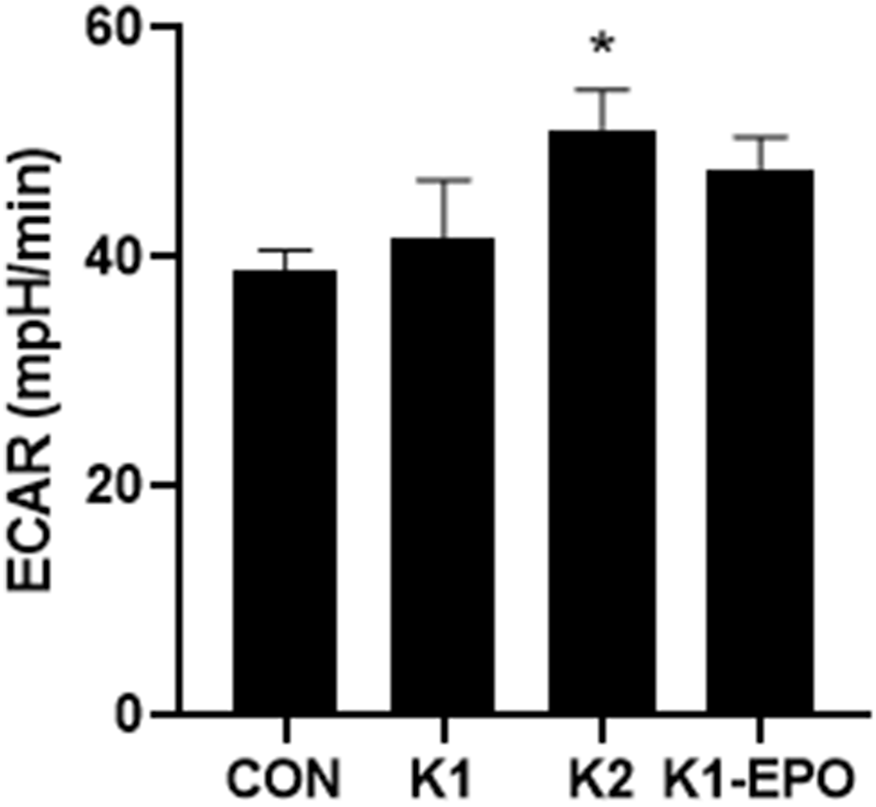

Supplementary Figure 3: Effect of vitamins K1 and K2 on extracellular acidification rate (ECAR). Cells were maintained in standard media supplemented with EtOH vehicle or 5 µg/ml K1, K2, or K1-EPO for > 3 passages. Cells were analyzed with the Seahorse XF Cell Mito Stress Test (Agilent). Basal ECAR values were calculated with WAVE software (Agilent) after normalization by DNA content. Bars represent mean ± SD of 3 technical replicates. *Significantly different from CON ($p < 0.05$) as measured by one-way ANOVA and Tukey post-test.

Supplementary Table 1: Ct values for vitamin K pathway genes.

See Supplementary File 1

Supplementary Table 2: Primer sequences for qPCR.

See Supplementary File 2
