# Supplementary figures and images for: Divergent effects of vitamins K1 and K2 on triple negative breast cancer cells
Source: Oncotarget. 2019 Mar 19;10(23):2292–305. doi: 10.18632/oncotarget.26765 (PMC6481349; doi:10.18632/oncotarget.26765)

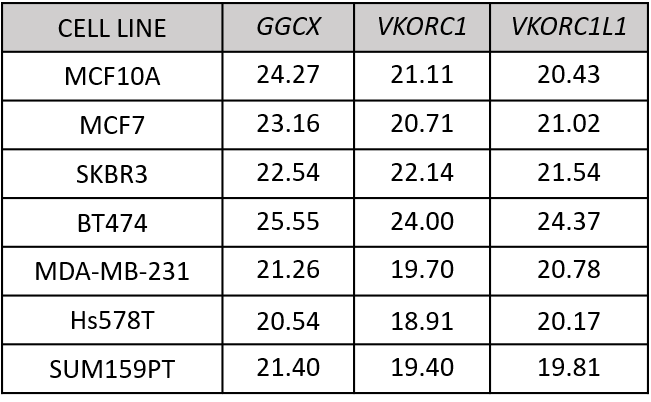


**SUPPLEMENTAL TABLE 1**

Ct Values for Vitamin K Pathway Genes

Supplement: Supplementary file 2 [file oncotarget-10-2292-s002.docx]

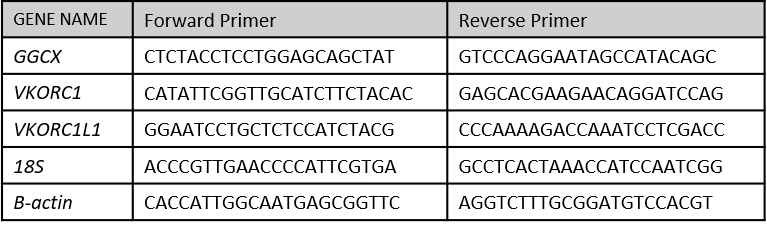


**SUPPLEMENTAL TABLE 2**

Primer Sequences

Supplement: Supplementary file 3 [file oncotarget-10-2292-s003.docx]
